# Endovascular intervention vs. microsurgery on the prognosis of anterior circulation blood blister-like aneurysm: A cohort study

**DOI:** 10.3389/fneur.2023.1103138

**Published:** 2023-03-23

**Authors:** Haibin Tan, Tian Zhang, Guangfu Huang, Zhili Li, Zhenyu Wang, Meixong Cheng, Ling Liu, Lingtong Liu

**Affiliations:** Department of Neurosurgery, Sichuan Provincial People's Hospital, University of Electronic Science and Technology of China, Chengdu, China

**Keywords:** intracranial aneurysm, anterior circulation, microsurgery, endovascular procedures, revascularization, cohort study

## Abstract

**Background:**

There are no universally acknowledged standardized treatment strategies for blood blister-like aneurysms (BBAs). This study compared the prognosis of patients with BBA who underwent craniotomy microsurgery vs. endovascular intervention.

**Methods:**

This retrospective cohort study included patients with BBA treated between September 2009 and August 2020 at Sichuan Provincial People's Hospital affiliated to the Sichuan Academy of Medical Science. Patients were divided into the microsurgery and endovascular groups. The preoperative Hunt-Hess grade and modified Fisher grade were collected. The intraoperative and postoperative complications (including intraoperative aneurysm rupture and hemorrhage, postoperative cerebral hemorrhage, and BBA recurrence) were recorded.

**Results:**

Seventy-two patients were included: 28 and 44 in the microsurgery and endovascular groups, respectively. Only the preoperative Fisher grade was different between the two groups (*P* = 0.041). The proportion of patients with good outcomes was lower in the microsurgery group (28.6%) than in the endovascular group (72.7%), and the mortality rate was higher in the microsurgery group (32.1%) than in the endovascular group (11.4%) (*P* < 0.05). After adjustment for the modified Fisher grade, the multivariable analysis showed that compared with craniotomy microsurgery, an endovascular intervention was associated with the prognosis of patients with BBA (OR = 0.128, 95%CI: 0.040–0.415, *P* < 0.001). The rate of complications (intraoperative hemorrhage, cerebral infarction, and recurrence) was higher in the microsurgery group than in the endovascular group.

**Conclusion:**

In patients with BBA, an endovascular intervention appears to be associated with a better prognosis compared with craniotomy microsurgery.

## Introduction

A blood blister-like aneurysm (BBA) is an aneurysmal bulge arising on the anterior or anteromedial wall of the intracranial segment of the internal carotid artery (ICA) and is not associated with branches of the ICA. The half-round-shaped aneurysm has a broad-base neck, thin wall, fragility, and blood blister-like morphology. BBA accounts for 0.9–6.5% of all intracranial aneurysms. BBA has a high tendency for rupture and hemorrhage, making clipping and interventions highly difficult (1, 2). Hence, the rates of recurrence, disability, and mortality are high (1, 2). There are still debates on the best treatments for BBA (3–5).

The pathogenesis of BBA includes the degeneration of the internal elastic lamina and the rupture of the tunica intima and media of the vascular wall, which is covered by fibrin tissues and extima. As there are blood clots but no inflammatory cell infiltration in the extima, similar to intracranial arterial dissections, BBA can be considered a pseudoaneurysm (6). Peschillo et al. (7) suggested that BBA and vertebral artery dissection share similarities, such as the patient population (young adults), instability of the vascular wall, and clinical characteristics. These pathological characteristics make BBA prone to enlarge to a saccular structure under the blood flow stress, leading to a relatively high risk of sudden rupture and hemorrhage during operation and poor outcomes.

Various treatment strategies have been tried for BBA, but no consensus has been reached on the best treatment (8). The current treatments can be classified mainly as treatments of the arterial aneurysm, hemodynamics modification, and repair of the parent artery wall. The techniques include microsurgical clipping, suturing of the parent artery, wrapping and strengthening of the aneurysm, bypass reconstruction, and aneurysm isolation. With the continuous advancement of interventional equipment and techniques, direct endovascular coils embolization, stent-assisted aneurysm embolization, and coated stent or dense mesh stenting with a higher metal coverage rate have also been extensively applied (8). Reconstruction and strengthening of the parent artery wall are critical for BBA treatment. Still, no universally acknowledged standardized treatment strategy is available yet, and only very few cases have been reported.

Therefore, this study investigated the prognosis of patients with BBA treated with craniotomy microsurgery vs. endovascular intervention.

## Materials and methods

### Availability of data and materials

The datasets used and/or analyzed for the present study are available from the corresponding author upon reasonable request.

### Study design and patients

This retrospective cohort study analyzed the clinical data of patients with BBA treated between September 2009 and August 2020 in the Neurosurgery Department of Sichuan Provincial People's Hospital affiliated to the Sichuan Academy of Medical Science. The study was approved by the ethics committee of Sichuan Provincial People's Hospital affiliated to the Sichuan Academy of Medical Science [approval #Lun Review (Research) 2022 No. 249]. The requirement for individual consent was waived by the committee because of the retrospective nature of the study.

The inclusion criteria were (1) proven BBA by preoperative digital subtraction angiography (DSA) and/or CT angiography (CTA), (2) available surgical findings, (3) clinical manifestations included sudden headache, and (4) plain CT scanning showed different degrees of subarachnoid hemorrhage (SAH) of the ICA cistern, suprasellar cistern, or lateral fissure cistern. The exclusion criteria were (1) did not undergo craniotomy microsurgery or endovascular intervention or (2) incomplete clinical data.

### Treatment

**Figure 1:**
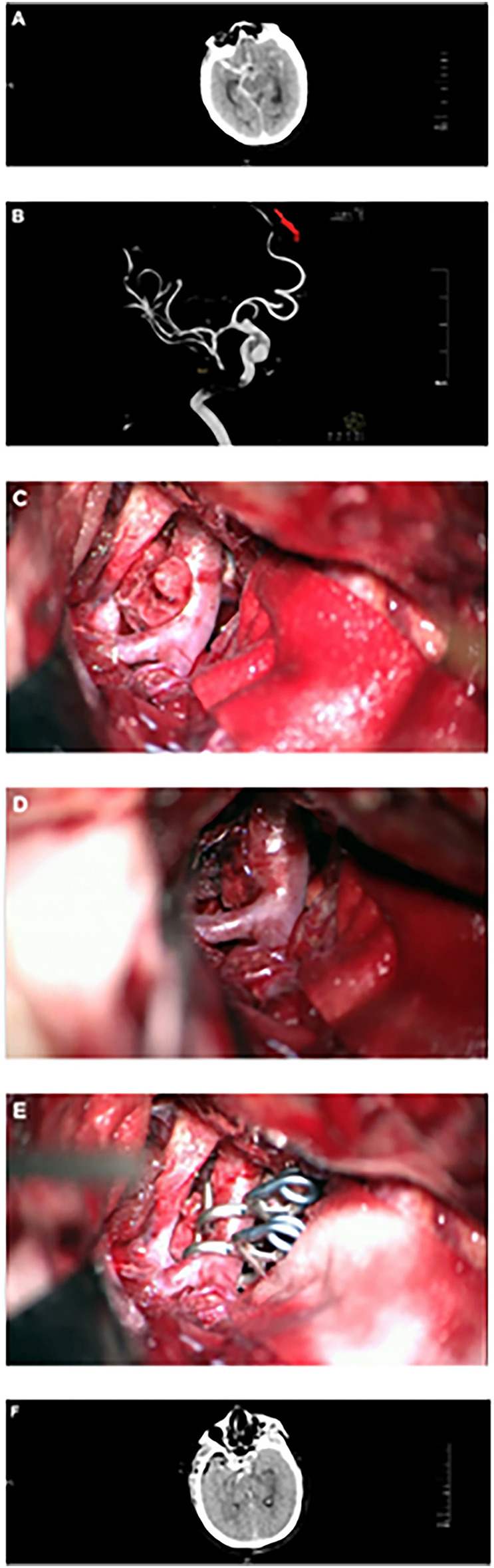
Craniotomy microsurgery for a typical case. **(A)** Head CT showed subarachnoid hemorrhage. **(B)** DSA showed BBA at the right terminal internal carotid artery. **(C)** Intraoperative microscopy showed BBA, mural thrombus, or fibrous cap formation at the top of the tumor. **(D)** Intraoperative microscopy showed BBA, mural thrombus or fibrous cap formation at the top of the tumor with intravascular turbulence. **(E)** BBA was clipped with a transvascular clamp arrangement. **(F)** Head CT showed obvious brain swelling 2 days after the operation.

According to the patient's condition and wishes, craniotomy microsurgery or endovascular intervention was performed. Figure 1 shows a typical case of BBA treated with craniotomy microsurgery, and Supplementary Figure 1 shows a typical case of BBA recurrence after craniotomy and treated with craniotomy microsurgery. Supplementary Figure 2 shows a typical case of BBA treated with endovascular intervention.

### Data collection

The demographic data (age and sex) of the patients were collected. The intraoperative and postoperative complications (including intraoperative aneurysm rupture and hemorrhage, postoperative cerebral hemorrhage, and BBA recurrence) were recorded. BBA recurrence refers to the recurrence of BBA after successful treatment. The modified Rankin scale (mRS) (8, 9) was used to assess neurological functions. The patients were divided into the good (mRS score of 0–2) and poor (mRS score of 3–6) prognosis groups (10). The preoperative Hunt-Hess grade (11) and modified Fisher grade (12) were collected.

### Statistical analysis

SPSS 20.0 (IBM Corp., Armonk, NY, USA) was used for statistical analysis. The Kolmogorov-Smirnov test was used to test the continuous data for normal distribution. The continuous data with a normal distribution were described as means ± standard deviations and analyzed using Student's *t*-test. The quantitative data with a skewed distribution were described as medians (P25, P75) and analyzed using the Mann-Whitney *U*-test. The categorical data were described as n (%) and analyzed using the chi-square test or Fisher's exact test. Multivariable logistic regression was used to assess the association between the surgical methods and BBA outcomes after adjustment for the Fisher grade. Two-sided *P*-values < 0.05 were considered statistically significant.

## Results

### Characteristics of the patients

Seventy-two patients were included: 28 in the microsurgery group and 44 in the endovascular group. The patients in the microsurgery group included 10 males and 18 females, and their mean age was 45.2 ± 11.6 years. The patients in the endovascular group included 18 males and 26 females, and their mean age was 44.8 ± 11.1 years. Only the preoperative Fisher grade was significantly different between the two groups (*P* = 0.041) (Table 1).

**Table 1:** Comparison of the general characteristics of 72 patients with BBA between the microsurgery and endovascular groups.

**General characteristics**	**Microsurgery group (*n* = 28)**	**Endovascular group (*n* = 44)**	***P***
Age (years)	45.2 ± 11.7	44.8 ± 11.1	0.883
**Sex**			0.645
Male	13 (46.4%)	18 (40.9%)	
Female	15 (53.6%)	26 (59.1%)	
**Preoperative HH grade**			0.173
I–II	12 (42.9%)	19 (43.2%)	
III	10 (35.7%)	22 (50.0%)	
IV	6 (21.4%)	3 (6.8%)	
**Preoperative fisher grade**			0.041
I–II	9 (32.1%)	25 (56.8%)	
III–IV	19 (67.9%)	19 (43.2%)	
Time from treatment to disease onset (d)	2.0 (1.0,6.8)	2.5 (1.5,7.0)	0.784
**Hyper-coagulation state**			0.147
Yes	17 (60.7%)	19 (43.2%)	
No	11 (39.3%)	25 (56.8%)	

DSA or CTA examination was performed for all patients, showing that the BBA was on the anterior wall of the superior segment of the clinoid segment of the ICA in 34 patients, on the medial wall of the terminal segment of the ICA in 27 patients, on the A1 segment of the anterior cerebral artery in nine patients, on the anterior communicating artery in one patient, and on the MCA in one patient. The mean diameter of the BBA was 3.4 ± 1.1 mm (range, 2–12 mm).

### Intraoperative and postoperative findings

In the microsurgery group, 22 patients were treated by direct clipping, three by aneurysm isolation and wrapping, one by direct aneurysm isolation, and two by bypassing and surgical isolation. Re-hemorrhage occurred in six patients after surgery (22.2%). CTA examination showed aneurysm recurrence or enlargement in seven patients (25.9%) who underwent interventional rescue therapy. Twenty-one patients (77.8%) had a cerebral infarction after the operation, and five patients (18.5%) had an evident midline shifting and were treated by decompressive craniectomy.

In the endovascular group, six patients were treated by single stent-assisted coil embolization, 26 by multiple stent dislocation overlapping technique-assisted coil embolization, and 12 by blood flow diverting devices. Re-hemorrhage occurred in 15 patients after surgery, and five patients underwent interventional rescue therapy for postoperative recurrence. Of nine patients (22.0%) who underwent blood flow diversion, two had intraoperative aneurysm rupture and hemorrhage, and no recurrence was found after the operation. Twenty-one patients (53.7%) had cerebral infarction after the operation, and 11 patients were treated with decompressive craniectomy. Postoperative monitoring of coagulation functions showed hyper-coagulability in 19 patients (46.3%).

### Postoperative complications and outcomes

The rate of complications (intraoperative hemorrhage, cerebral infarction, and recurrence) was significantly higher in the microsurgery group than in the endovascular group. In the microsurgery group, eight, 11, and nine patients had mRS scores of 0–2, 3–5, and 6 (death) at discharge. In the microsurgery group, at discharge, 32, seven, and five patients had mRS scores of 0–2, 3–5, and 6 (death), respectively. The proportion of patients with good outcomes was significantly lower in the microsurgery group (29.6%) than in the endovascular group (73.2%), and the mortality rate was significantly higher in the microsurgery group (33.3%) than in the endovascular group (12.2%) (*P* < 0.05) (Table 2).

**Table 2:** Comparison of complications, postoperative recurrence, recurrence during follow-up, and outcomes at discharge in the 72 patients in the microsurgery and endovascular groups.

	**Microsurgery group (*n* = 28)**	**Endovascular group (*n* = 44)**	***P***
**Complication**			
Intraoperative hemorrhage	18 (66.7%)	17 (41.5%)	0.042
Infarction	21 (77.8%)	22 (53.7%)	0.044
Recurrence	10 (37.0%)	5 (12.2%)	0.016
**mRS score**			
0–2	8 (28.6%)	32 (72.7%)	< 0.001
3–5	11 (39.3%)	7 (15.9%)	0.033
6 (death)	9 (32.1%)	5 (11.4%)	0.035

mRS, modified Rankin scale.

After adjustment for the modified Fisher grade, the multivariable analysis showed that endovascular intervention resulted in better outcomes for patients with BBA compared with craniotomy microsurgery (OR = 0.128, 95% CI: 0.040–0.415, *P* < 0.001) (Table 3).

**Table 3:** Results of the multivariable logistic regression.

	**OR (95%CI)**	***P***
**Surgical method**		
Microsurgery group	Reference	Reference
Endovascular group	0.128 (0.040–0.415)	< 0.001

## Discussion

The present study showed that endovascular intervention achieved better outcomes than craniotomy microsurgery in patients with BBA. Indeed, compared with surgical treatment, endovascular intervention appears to be associated with lower recurrence and mortality rates and better outcomes.

The imaging diagnosis of BBA is challenging, and small BBAs are sometimes very difficult to detect by the first DSA or CTA. In the present study, two patients were diagnosed with saccular aneurysms by preoperative CTA, while intraoperative findings showed “pseudocystic” BBA. BBA consists of only a layer of fibrin membrane and hematoma, which is prone to spontaneous rupture and hemorrhage to induce the rapid enlargement of the aneurysm, showing a cystic morphology. Therefore, BBA and saccular aneurysms are relatively difficult to be distinguished by imaging examinations (7).

The present study demonstrated that the incidence of intraoperative rupture and hemorrhage was higher in the microsurgery group than in the endovascular group. Of the 27 patients who underwent craniotomy microsurgery, 18 had intraoperative rupture and hemorrhage, which occurred when dissecting the aneurysm and were possibly caused by an avulsion of the BBA and ICA, as well as the slipping of the aneurysm neck from the clamp during the procedures. Six patients had a hemorrhage recurrence shortly after surgery, which could be associated with twisting and slipping of the aneurysm neck, incomplete clipping, or insufficient clipping of the parent artery. The perioperative re-hemorrhage rate was < 4% for saccular aneurysms (13). BBA has a higher probability of perioperative re-rupture and hemorrhage, indicating BBA can re-grow (7). Of 41 patients in the endovascular group, 17 had intraoperative rupture and hemorrhage, and five patients had a hemorrhage recurrence shortly after the procedure, which could be associated with the metal coverage rate of the stent, immediate intraoperative embolization degree, and the postoperative use of dual antiplatelet drugs.

The incidence of postoperative cerebral infarction was high in the patients with BBA in this study, irrespective of treatment than in patients with saccular aneurysms, which could be associated with severe vasospasm and hypercoagulability (14). The ratio of cerebral infarction was significantly higher in the microsurgery group than in the endovascular group, which could be associated with the early use of anti-platelet drugs in the endovascular group. The severity of postoperative cerebral infarction could also influence the outcomes of the neurological functions of the patients.

Treating BBA using the single or multiple stents dislocation overlapping techniques and relatively soft coils embolization can reduce the mesh ratio of the stent to prevent the coils from slipping off, thus achieving effective and dense filling and reducing the risk of rupture. In addition, this treatment could also increase the metal coverage rate to exert vascular reconstruction and blood flow diverting effects, thus reducing the perioperative recurrence rate and improving the Raymond stage in DSA follow-up (15). Compared with other endovascular treatments, treatment using blood flow diverting devices has a higher BBA occlusion rate, which was not accompanied by higher complication incidence, and the clinical outcomes are comparable (16, 17). The major mechanism of blood flow diverting devices involves the use of dense mesh stent to reconstruct the local blood flow and direct the blood in the parent artery, thus reducing the impact of local blood flow on the aneurysm wall, significantly reducing the blood flow in the aneurysm, and leading to thrombosis and occlusion in the aneurysm. Blood flow-diverting devices mainly reconstruct the parent artery without directly touching the fragile BBA.

This study has limitations. The patients were from a single center, and the sample size was small. Two treatment strategies were compared, but the reasons and indications for selecting one over the other were not readily available in the patient charts. Even though the two groups appear similar, there were probably differences in some characteristics that were not analyzed. Indeed, because of the study's retrospective nature, only the data available in the charts could be analyzed. Additional studies are still necessary to determine the optimal treatment of BBA.

## Conclusion

Timely treatment complications and close imaging follow-up are very important for improving the outcomes of patients with BBA. Compared with surgical treatment, the endovascular treatment appears to have a lower incidence of complications and a lower mortality rate, which could also lead to better neurological outcomes.

## Data availability statement

The original contributions presented in the study are included in the article/Supplementary material, further inquiries can be directed to the corresponding author.

## Ethics statement

The studies involving human participants were reviewed and approved by the Ethics Committee of Sichuan Provincial People's Hospital affiliated to the Sichuan Academy of Medical Science [#Lun Review (Research) 2022 No. 249]. Written informed consent for participation was not required for this study in accordance with the national legislation and the institutional requirements.

## Author contributions

Chief physician HT performed craniotomy for some of the cases in the paper, and the case information and data were sorted and analyzed. Three chief physicians, GH, ZL, and ZW, performed craniotomy and microsurgery in some cases. Attending physicians LingL and LingtongL participated in part of the craniotomy. Chief Physician TZ and attending physician MC performed all the endovascular interventions. All authors read and approved the final manuscript.
