# Supplementary material for: Endovascular intervention vs. microsurgery on the prognosis of anterior circulation blood blister-like aneurysm: A cohort study
Source: Front Neurol. 2023 Mar 23;14:1103138. doi: 10.3389/fneur.2023.1103138 (PMC10076783; doi:10.3389/fneur.2023.1103138)
Supplement: Supplementary file 1 [file Table_1.DOCX]

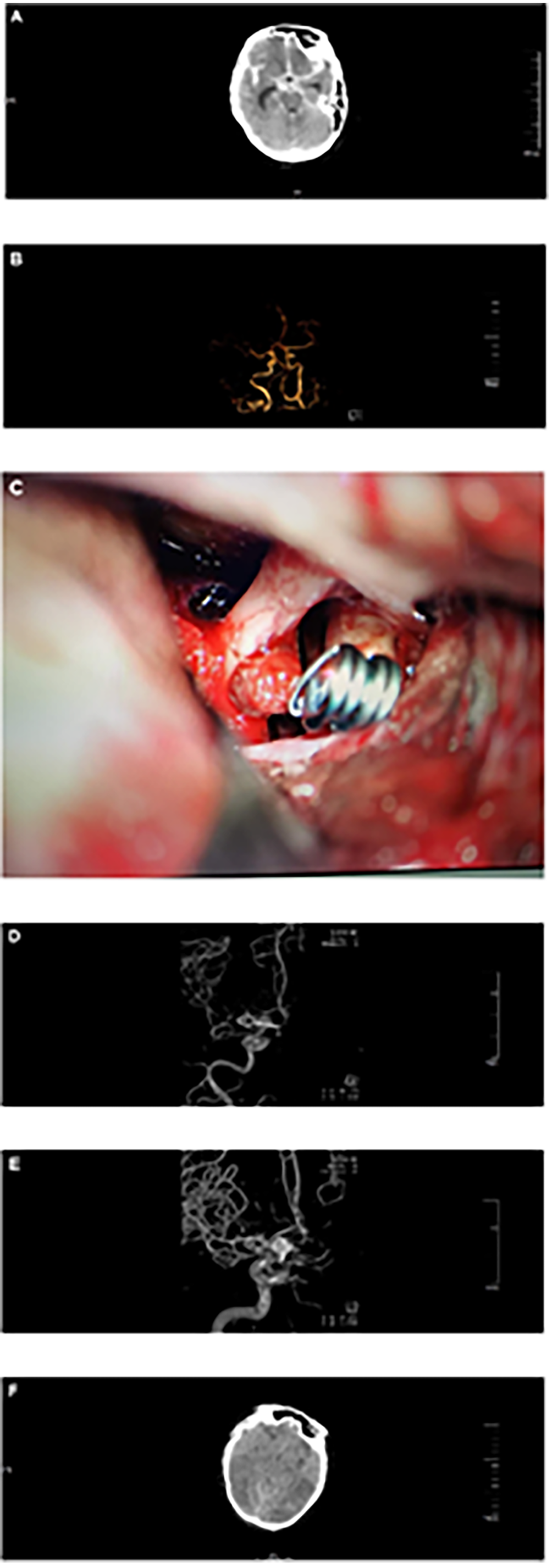


**Figure S1. A typical case recurred after craniotomy and were treated with interventional therapy.** A: Head CT showed subarachnoid hemorrhage; B: Preoperative CTA revealed a "cystic" aneurysm at the end of the right internal carotid artery; C: A "pseudocystic" blood blister-like aneurysm was found during the operation; D: One week after surgery, head CT showed SAH again, and DSA showed recurrence of wide-necked aneurysm in the communicating segment of the right internal carotid artery with vasospasm; E:Embolization of the tumor sac with LVIS stent-assisted coil; F: Postoperative CT showed multiple cerebral infarction in both hemispheres.


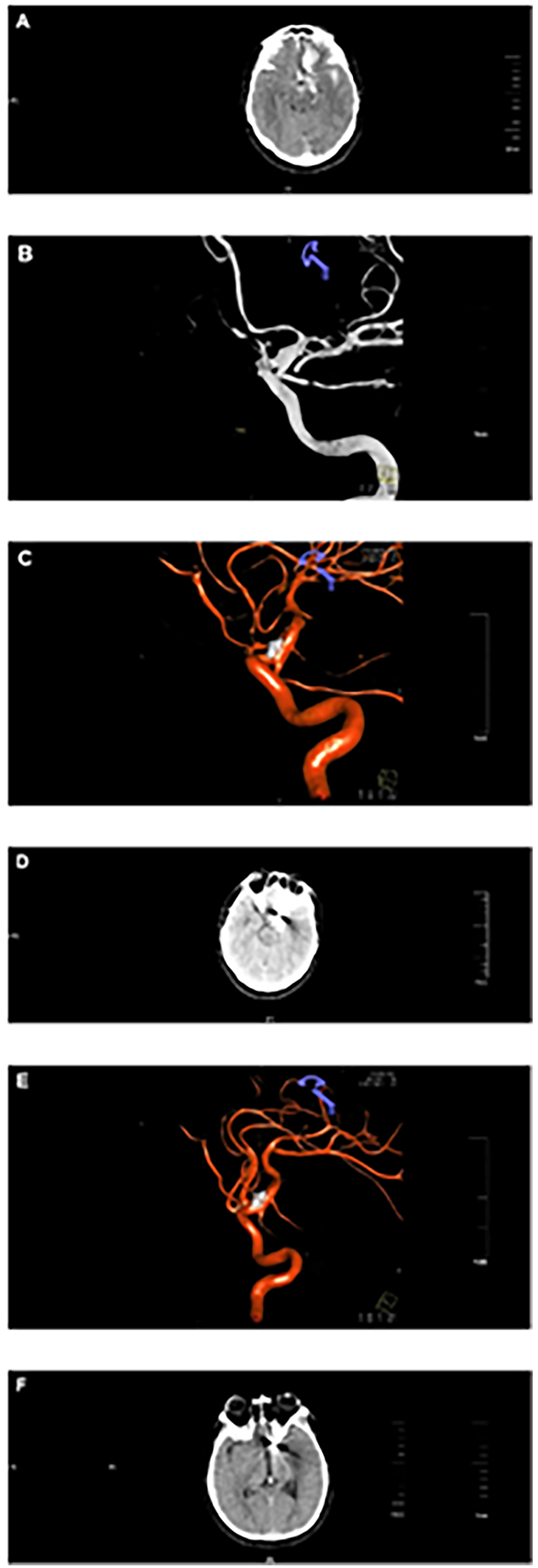


**Figure S2. Endovascular intervention for a typical case.** A: Head CT showed subarachnoid hemorrhage; B: DSA showed BBA in the communicating segment of the left internal carotid artery and obvious vasospasm in the cerebral artery; C: Endovascular stent assisted coil embolization; D: Cranial CT scan showed brain swelling 1 day after operation; E: DSA double volume reconstruction at 12 months after operation showed that the parent artery was unobstructed and there was no recurrence of BBA; F: CT follow-up 12 months after operation showed multiple focal cerebral infarction and encephalomalacia.
